# Inhibition of diastatic yeasts by *Saccharomyces* killer toxins to prevent hyperattenuation during brewing

**DOI:** 10.1128/aem.01072-24

**Published:** 2024-09-12

**Authors:** Victor Zhong, Nicholas Ketchum, James K. Mackenzie, Ximena Garcia, Paul A. Rowley

**Affiliations:** 1 Department of Biological Sciences, University of Idaho, Moscow, Idaho, USA; 2 Rhinegeist Brewery, Cincinnati, Ohio, USA; 3 Institute for Modeling Collaboration and Innovation, University of Idaho, Moscow, Idaho, USA; The Pennsylvania State University, University Park, Pennsylvania, USA

**Keywords:** diastatic, *Saccharomyces cerevisiae*, yeast, brewing, killer yeast, killer toxins

## Abstract

Secondary fermentation in beer can result in undesirable consequences, such as off-flavors, increased alcohol content, hyperattenuation, gushing, and the spontaneous explosion of packaging. Strains of *Saccharomyces cerevisiae var. diastaticus* are a major contributor to such spoilage due to their production of extracellular glucoamylase enzyme encoded by the *STA1* gene. *Saccharomyces* yeasts can naturally produce antifungal proteins named “killer” toxins that inhibit the growth of competing yeasts. Challenging diastatic yeasts with killer toxins revealed that 91% of strains are susceptible to the K1 killer toxin produced by *S. cerevisiae*. Screening of 192 killer yeasts identified novel K2 toxins that could inhibit all K1-resistant diastatic yeasts. Variant K2 killer toxins were more potent than the K1 and K2 toxins, inhibiting 95% of diastatic yeast strains tested. Brewing trials demonstrated that adding killer yeast during a simulated diastatic contamination event could prevent hyperattenuation. Currently, most craft breweries can only safeguard against diastatic yeast contamination by good hygiene and monitoring for the presence of diastatic yeasts. The detection of diastatic yeasts will often lead to the destruction of contaminated products and the aggressive decontamination of brewing facilities. Using killer yeasts in brewing offers an approach to safeguard against product loss and potentially remediate contaminated beer.

**IMPORTANCE:**

The rise of craft brewing means that more domestic beer in the marketplace is being produced in facilities lacking the means for pasteurization, which increases the risk of microbial spoilage. The most damaging spoilage yeasts are “diastatic” strains of *Saccharomyces cerevisiae* that cause increased fermentation (hyperattenuation), resulting in unpalatable flavors such as phenolic off-flavor, as well as over-carbonation that can cause exploding packaging. In the absence of a pasteurizer, there are no methods available that would avert the loss of beer due to contamination by diastatic yeasts. This manuscript has found that diastatic yeasts are sensitive to antifungal proteins named “killer toxins” produced by *Saccharomyces* yeasts, and in industrial-scale fermentation trials, killer yeasts can remediate diastatic yeast contamination. Using killer toxins to prevent diastatic contamination is a unique and innovative approach that could prevent lost revenue to yeast spoilage and save many breweries the time and cost of purchasing and installing a pasteurizer.

## INTRODUCTION

The rising popularity of craft beers and the growth of the craft brewing industry means that more beer is being produced in facilities lacking pasteurization (1). Pasteurization stabilizes beer against contamination by spoilage organisms, including yeast and bacteria. The shift away from pasteurization is likely because of the high capital costs of pasteurization equipment and increased energy and water usage (2, 3). In addition, the dominant beer styles produced by the craft brewing industry are negatively affected by the high temperature of the pasteurization process. Beer styles that have been aggressively hopped post-boil, such as India Pale Ales (IPAs), will suffer from excessive exposure to oxygen when pasteurized (4). This treatment is perceived to increase the rate of staling and can lead to off-putting flavors described as papery, wet, cardboard-like, leathery, or even “catty.” This means that many breweries will avoid pasteurizing as it will cause the degradation of delicate hop aromas. This aversion to pasteurization can increase the risk of spoilage in craft breweries. Although the antimicrobial properties of hops can protect beers from bacterial spoilage, yeasts are more resistant and represent a more problematic spoilage organism without pasteurization.

One group of yeasts that cause spoilage in craft breweries are *Saccharomyces cerevisiae* strains that express the *STA1* gene to produce an extracellular glucoamylase enzyme. These strains have been referred to as diastatic yeasts, a name that is derived from diastase, an alternative nomenclature for amylase. Diastatic yeasts are an evolutionarily related group of *S. cerevisiae* strains used commercially to produce high-gravity Belgian-style beers (5, 6). Diastatic yeasts are unique because the *STA1* gene allows the hydrolysis of long-chain polysaccharides such as dextrin and starch. In non-Belgian-style beer, these carbohydrates remain after the primary fermentation has consumed simple di- and monosaccharides created in the mashing process. Dextrins and starches are usually unavailable to most commercial brewing strains as they lack the appropriate hydrolytic enzymes to break the glycosidic linkages between carbohydrate monomers. The *STA1* gene evolved due to a fusion of *FLO11* and *SGA1* (7, 8). The gene fusion resulted in a chimeric protein with the N-terminus of the *FLO11* gene joined to almost the complete open reading frame of the *SGA1* glucoamylase. The 5′ end of *FLO11* fused to *SGA1* enabled the transport of Sta1 into the extracellular milieu, where it can hydrolyze residual dextrin and starch to glucose monomers. The resulting glucose is used to prolong fermentation (usually after packaging), referred to as over-, super-, or hyperattenuation. Hyperattenuation results in the overproduction of CO_2_ and alcohol, imparting off flavors and promoting “gushing” and the explosion of containers. The *STA1* gene is also part of a family undergoing gene duplications and translocations, creating the paralogs *STA2* and *STA3* (9). One of the few publicized examples of a major diastatic yeast contamination resulted in the recall of $2 million of packaged beer by Left-hand Breweries and was the subject of a $6 million lawsuit (10). Significantly, the overall occurrence of spoilage by diastatic yeasts in Europe increased between 2008 and 2017 and is an important problem for the brewing industry worldwide (11, 12).

Good hygiene, strain husbandry, and monitoring practices can reduce the likelihood of contamination by diastatic yeasts. However, viable cell counts on agar-based media can be somewhat unreliable and take days after sampling to identify contaminants (13). The gold-standard molecular methods for rapidly detecting the *STA1* gene by PCR require specialized equipment, reagents, and personnel to perform and interpret these technically demanding assays (14). Even if diastatic yeast contamination is detected, the primary course of action in a brewery without a pasteurizer is the destruction of the contaminated product. Therefore, there is an urgent need to develop cost-effective technologies that actively prevent or remediate contamination by diastatic yeasts.

Killer yeasts can produce extracellular proteinaceous killer toxins that inhibit the growth of competing species of fungi (15–18). Many studies have shown the effectiveness of killer yeasts in preventing spoilage of fruits, silage, and wine fermentation (19–26). These successes have led to the application of certain fungal species and genetically engineered crops as biological controls in agricultural processes (27–30). *Saccharomyces* yeasts were some of the first species identified as producing killer toxins (31), and surveys have estimated that many strains of *S. cerevisiae* are killer yeasts (32, 33). Killer toxin expression often depends on cytoplasmic double-stranded RNAs (dsRNAs) replicated and encapsidated by viruses of the family *Totiviridae* (34). To date, nine dsRNA-encoded killer toxins are produced by different strains of *S. cerevisiae* (K1, K2, K28, and Klus) and *S. paradoxus* (K62, K1L, K21, K74, and K21/K66) (35–42). At least two functional genome-encoded killer toxins exist in *S. cerevisiae* (KHR and KHS) (43, 44). These killer toxins share little primary sequence homology and can target susceptible cells by different mechanisms, such as by disrupting cell membranes (K1, K1L, and K2) or by arresting the cell cycle (K28) (45, 46). The antifungal activities of killer toxins are generally limited to closely related species, and there is evidence of widespread resistance across different yeast lineages, which has limited their potential as broad-spectrum antifungals (47, 48). However, despite notable resistance to killer toxins, some studies have shown that the canonical killer toxins of *Saccharomyces* yeasts can successfully inhibit specific human and agricultural pathogens (49–53).

This study demonstrates that killer toxins from *Saccharomyces* yeasts have potent antifungal activity against different strains of diastatic yeasts. After screening an extensive collection of *Saccharomyces* killer yeast, diastatic strains resistant to canonical killer toxins were found to be susceptible to a non-canonical variant of the K2 toxin named K2v. As proof-of-principle for applying killer yeasts to control diastatic contamination, a K2 killer yeast strain was able to prevent hyperattenuation in an industrial-scale fermentation with no adverse effect on the final gravity. This work provides a framework for using killer yeasts in the craft brewing industry to prevent future losses and lawsuits.

## RESULTS

**Fig 1:**
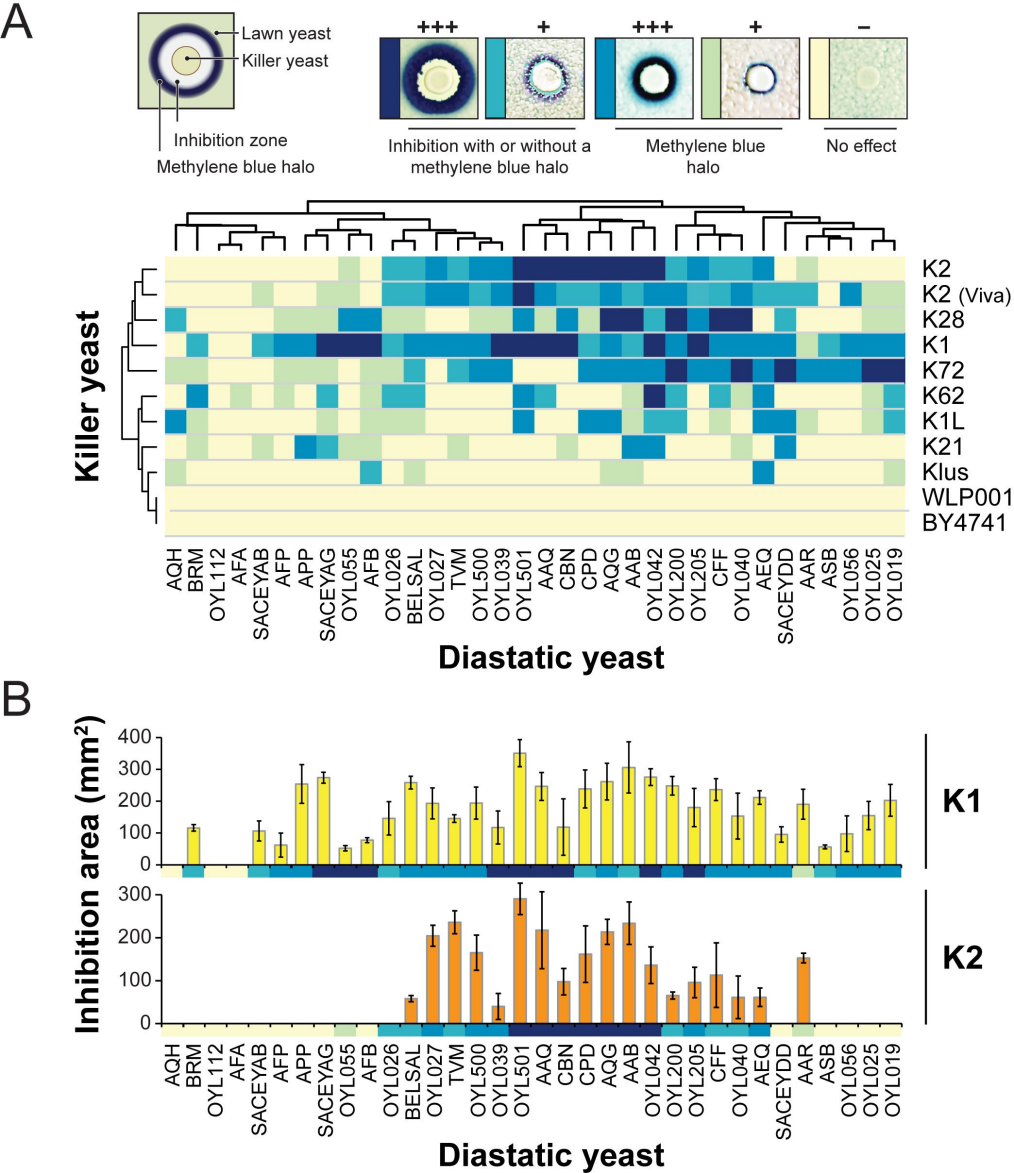
Diastatic yeasts are susceptible to canonical killer toxins produced by *Saccharomyces* yeasts. (**A**) Killer toxin activity against diastatic yeasts was qualitatively assessed based on the presence and size of growth inhibition zones and methylene blue staining around killer yeasts as diagrammed (top left). Darker colors on the cluster diagram represent a more prominent killer phenotype, with yellow indicating no detectable killer phenotype (top right). The non-killer yeast strains *S. cerevisiae* BY4741 (a laboratory strain) and WLP001 (a brewing yeast) were used as negative controls. Images in the key were reproduced from (41) and (51). (**B**) The antifungal activity of K1 and K2 against diastatic yeasts was quantified by measuring the total zone of growth inhibition (*n* = 3). Error bars are standard deviation. Data from panel A are represented across each x-axis for comparison.

To determine the susceptibility of diastatic (*STA1*+) strains of *S. cerevisiae* to killer toxins, 34 diastatic strains were challenged by *Saccharomyces* yeasts expressing eight different canonical killer toxins (K1, K1L, K2, K21/K66, K28, K62, K74, and Klus) (Fig. 1A). Zones of growth inhibition and halos of methylene blue surrounding the killer yeast indicated the susceptibility of diastatic yeasts to the antifungal activities of killer toxins. Whereas zones of growth inhibition showed no growth of diastatic yeasts, methylene blue halos likely resulted from the initial growth of diastatic yeasts, followed by cell death due to sustained killer toxin exposure. Specifically, loss of viability results in the oxidation of methylene blue present in diastatic yeast cells and the appearance of blue-stained cells. The extent of growth inhibition was first qualitatively scored according to the degree of growth inhibition and methylene blue staining using a high-throughput plating assay (Fig. 1A; Table S1). Of all the canonical killer toxins assayed, K1 was judged to be the most inhibitory to diastatic yeasts and could prevent the growth of 91.2% of the diastatic strains tested. K2 could inhibit the growth of 58.8% of diastatic strains and, after K1, produced the largest zones of growth inhibition with methylene blue halos. The potency of K1 and K2 against diastatic yeast was further confirmed by quantitatively comparing the area of killer toxin inhibition against all diastatic strains tested (Fig. 1B). This analysis again found that K1 was the most effective at inhibiting diastatic yeasts. Overall, the quantitative analysis agreed with the K1 qualitative assay and had only two false positives (OYL055 and OYL026) across the K2 dataset. Overall, K1 was significantly more potent than K2, with an average area of growth inhibition of 175.5 mm^2^ (SD; 84.5), whereas the average for K2 was 87.6 mm^2^ (SD; 92.9) (Student’s two-tailed *t*-test, *P* < 0.05). This demonstrated that diastatic yeasts are particularly sensitive to the K1 killer toxin produced by *S. cerevisiae*.

**Fig 2:**
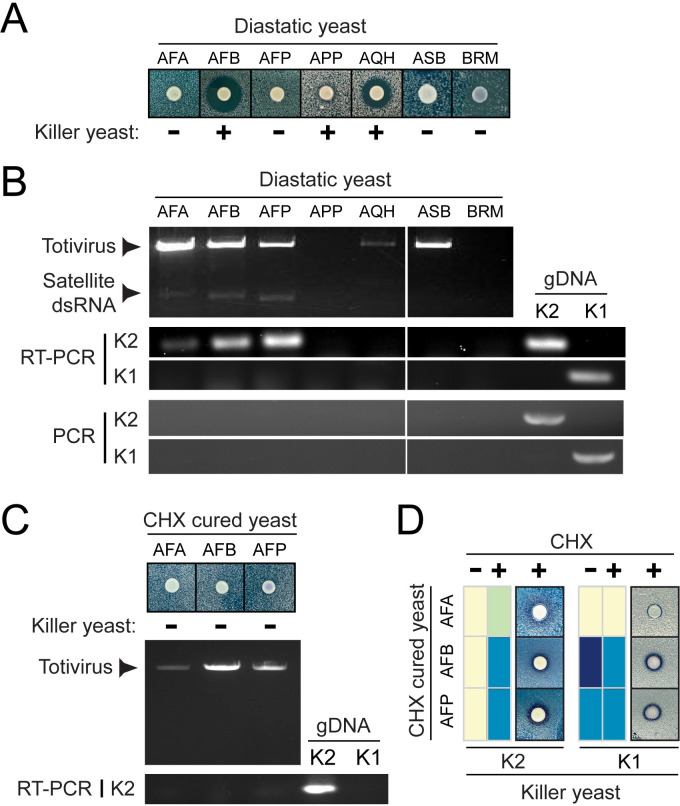
K2 killer toxin production by diastatic yeasts causes K2 resistance. (**A**) Killer toxin production by diastatic yeasts resistant to K1 and K2 killer toxins. (+) indicates a diastatic killer yeast capable of inhibiting the growth of *S. cerevisiae* BY4741. (−) indicates a non-killer yeast. (**B**) The extraction and analysis of dsRNAs from the diastatic yeasts by cellulose chromatography and gel electrophoresis. The detection of K1 or K2 genes by RT-PCR or PCR and using dsRNA or genomic DNA as templates. Genomic DNA extracted from *S. cerevisiae* with K1 or K2 genes integrated into the genome was used as a positive control. (**C**) Exposure to cycloheximide (CHX) was used to cure diastatic strains of the killer phenotype due to the loss of dsRNA satellites as assayed by cellulose chromatography and RT-PCR. (**D**) Curing of dsRNAs resulted in susceptibility to K2 but not K1 as assayed on agar. Killer toxin activity against cured (CHX +) and wild-type (CHX −) diastatic yeasts was qualitatively assessed based on the presence and size of growth inhibition zones and methylene blue staining around either K1 or K2 killer yeasts (as described in Fig. 1A).

Killer toxin production by *Saccharomyces* yeasts is accompanied by immunity to the mature toxin. To determine whether the killer toxin-resistant diastatic yeasts had gained immunity due to killer toxin production, three K1-resistant diastatic yeasts and an additional ten strains resistant to K2 were used to challenge three lawns of *S. cerevisiae* known to be susceptible to K1 or K2. Only three diastatic strains were identified as killer yeasts (APP, AQH, and AFB) (Fig. 2A; Fig. S1). To determine whether killer toxin production was due to viruses and associated dsRNA satellites, each of the 14 killer toxin-resistant diastatic yeasts was subjected to analysis by cellulose chromatography to purify dsRNAs. This analysis revealed that five strains contained dsRNAs with sizes the same as totiviruses (~4.6 kb), and three strains harbored an additional satellite dsRNA (~1.5 kb) (Fig. 2B; Fig. S1). Using total nucleic acid samples, reverse transcriptase PCR (RT-PCR) was used to detect the K2 killer toxin gene in the strains AFA, AFB, and AFP (Fig. 2B). K1 was not detected in any strains assayed by RT-PCR, and PCR alone could not amplify K1 or K2, indicating that the DNA genome does not encode these killer toxin genes (Fig. 2B). Exposure to cycloheximide was used to cure the satellite dsRNAs from the killer yeasts AFA, AFB, and AFP, as determined by cellulose chromatography and RT-PCR (Fig. 2C). This curing treatment resulted in the loss of killer toxin production and susceptibility to K2, with K1 susceptibility remaining unchanged (Fig. 2D). These data show that while K1 resistance of diastatic yeasts was independent of dsRNAs, K2 resistance was due to the presence of M2 dsRNA satellites.

**Fig 3:**
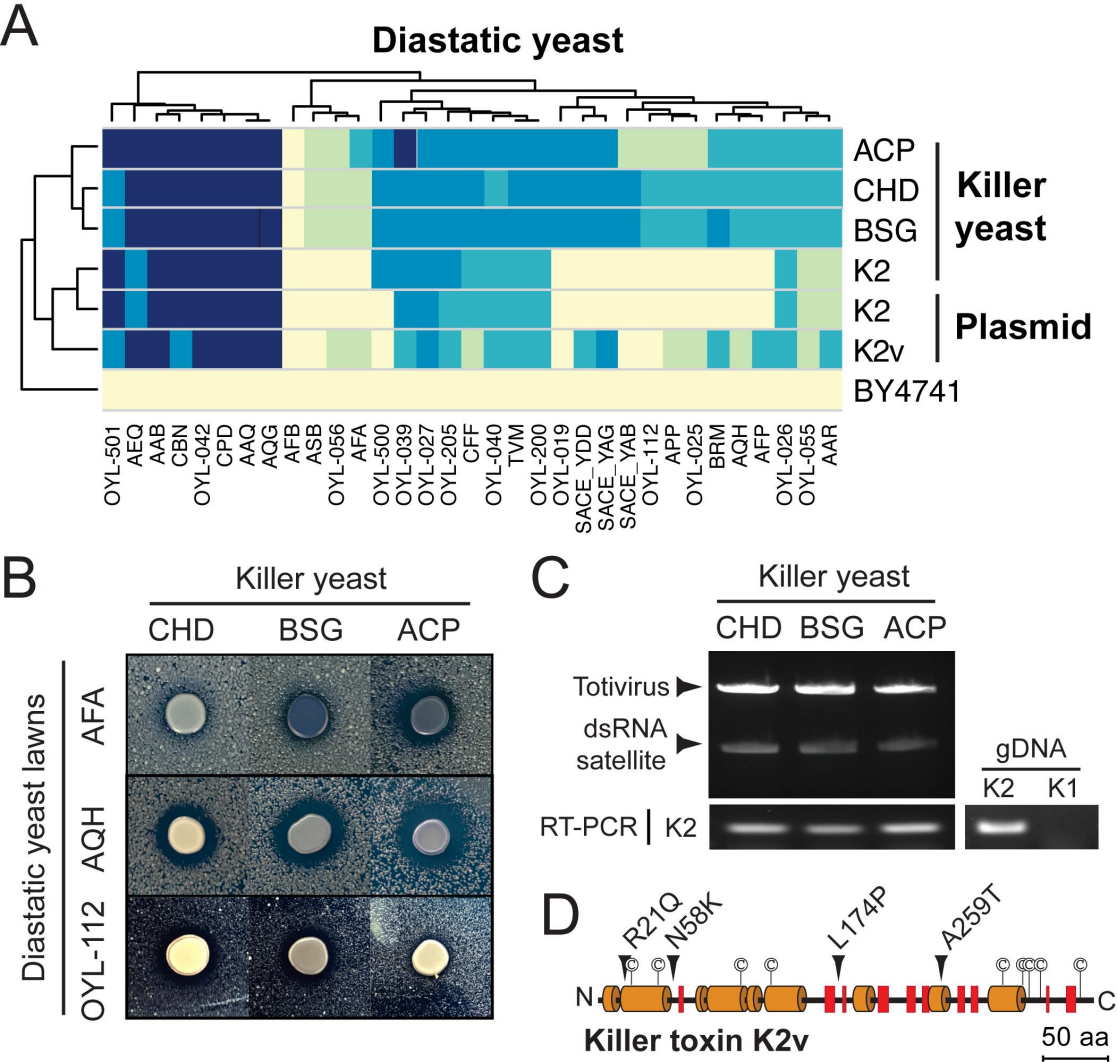
A novel killer toxin named K2v can inhibit the growth of diastatic yeasts resistant to K1 and K2 killer toxins. (**A**) Killer toxin activity against diastatic yeasts was qualitatively assessed based on the presence and size of growth inhibition zones and methylene blue staining around killer yeasts (as described in Fig. 1). Killer toxins were expressed by wild-type killer yeasts or killer toxin genes maintained on plasmids and expressed in the non-killer *S. cerevisiae* strain BY4741. K2v was cloned from strain ACP. The non-killer yeast *S. cerevisiae* strain BY4741 was used as a negative control. (**B**) Representative agar plate killer assays of the sensitivity of K1- and K2-resistant diastatic yeasts to three novel killer yeasts expressing K2v. (**C**) Novel killer yeasts harbor totiviruses and satellite dsRNAs, as confirmed by RT-PCR. Genomic DNA extracted from *S. cerevisiae* with K1 or K2 genes integrated into the genome was used as a positive control. (**D**) A linear representation of the secondary structure of the K2v killer toxin from strain ACP. Orange cylinders and red rectangles represent α-helices and β-sheets, respectively.

Diastatic yeast strains were resistant to K1 and K2 killer toxins (AFA, AQH, and OYL-112) and K1 and K74 (AFA and OYL-112). The diastatic strain OYL-112 was resistant to all canonical killer toxins. Therefore, 192 previously identified and uncharacterized *S. cerevisiae* killer yeasts were screened to determine whether they could inhibit the growth of killer toxin-resistant diastatic yeasts (33). In total, 32 killer yeasts were able to cause growth inhibition of K1 and K2-resistant diastatic yeast (Table S2). Three strains of killer yeasts (CHD, BSG, and ACP) were judged the most effective at inhibiting the growth of killer toxin resistant diastatic yeasts (Fig. 3A). These killer yeasts also inhibited the growth of all other diastatic yeast strains except the K2-resistant diastatic strain AFB (Fig. 3B). These three novel killer yeasts were analyzed for dsRNAs using cellulose chromatography, which found that all three harbored totiviruses and satellite dsRNAs. RT-PCR confirmed that these strains were K2 killer yeast (Fig. 3C). This result was surprising as this novel K2 variant could inhibit strains AFA, AQH, and OYL-112, which were all resistant to the canonical K2 toxin (Fig. 1).

Purification and sequencing of the dsRNAs from *S. cerevisiae* strains CHD, BSG, and ACP confirmed that all three strains contained satellite dsRNAs with K2 killer toxin genes. These K2 genes had four non-synonymous mutations compared to canonical K2 (Fig. 3D). To distinguish this mutant toxin from canonical K2, it will be referred to as K2-variant (K2v) and the satellite dsRNA as M2v. K2v and K2 genes were introduced into a plasmid for expression in a non-killer laboratory strain of *S. cerevisiae* to directly compare the effect of the observed non-synonymous mutations on the spectrum of killer toxin activity. Comparing the galactose-induced expression of K2 and K2v from a high copy plasmid, it was found that K2v had a broader spectrum of antifungal activity that could inhibit 78% of diastatic yeasts compared to K2, which inhibited only 50% (Fig. 3A). Galactose-induced expression of K2 was almost identical to the wild-type K2 killer yeast, but plasmid-expressed K2v inhibited less diastatic yeasts than the K2v killer yeasts CHD, BSG, and ACP (Fig. 3A). Plasmid-expressed K2v could not inhibit the diastatic K2 killer yeast AFB that harbored an M2 satellite dsRNA, suggesting that K2 immunity function could protect this strain from the K2v killer toxin. Surprisingly, K2v could inhibit the K2-resistant diastatic strains AFA and AFP that also harbored M2, indicating that K2 in these strains is insufficient for K2v immunity. Overall, K2v is characterized as a variant K2 killer toxin with a broad-spectrum activity against diastatic yeast compared to canonical K2. Mutations in K2v likely caused changes in the killer toxin spectrum of activity and immunity that could inform the future development and application of K2 against diastatic yeasts.

**Fig 4:**
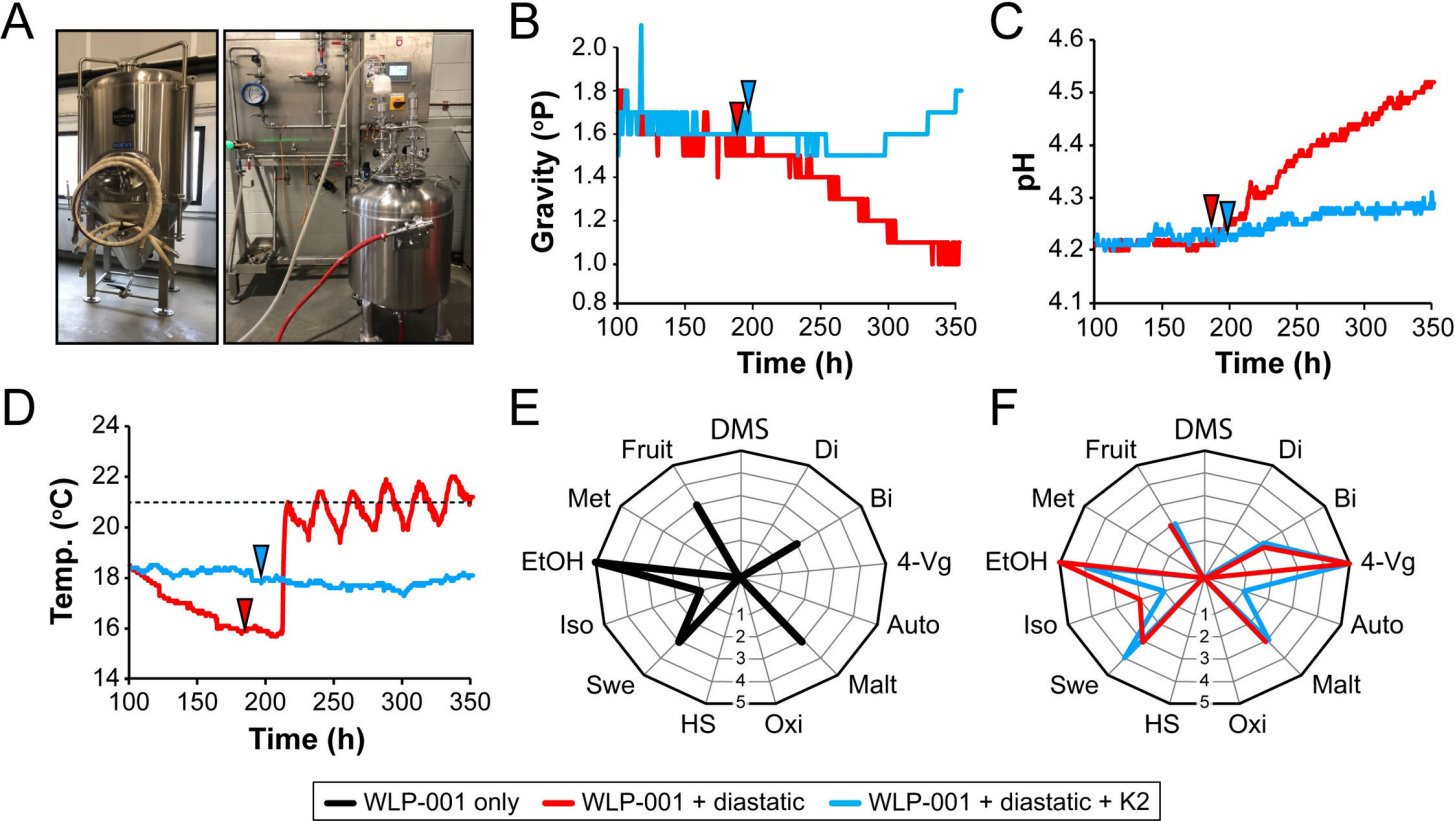
Killer yeasts can prevent hyperattenuation in fermentation trials. (**A**) Two ten-hectoliter (1,000 L) pioneer fermentation vessels were used for the fermentation trials (left panel) with the brewing strain WLP-001, diastatic strain Belle Saison, and the K2 killer yeast strain Viva. A 100 L Esau Huber microprop Yeast propagation plant was used to grow brewing and diastatic yeast strains (right panel). The specific gravity (**B**), pH (**C**), and temperature (**D**) of the brewing trials were monitored for ~14 days with (blue line) or without (red line) the addition of a K2 killer yeast (Viva). The dashed line in (**D**) represents the set point of 21°C for cooling. Arrows indicate the addition of diastatic yeast after 7–8 days of fermentation with (blue arrow) or without (red arrow) the addition of the K2 killer yeast strain (Viva). A spider diagram depicting tasting notes from the fermentation of (**E**) WLP-001 and (**F**) WLP-001 and diastatic yeast with (blue line) or without (red line) the addition of a K2 killer yeast strain (Viva). Sensory characteristics are judged on a 10-point scale from absent (0) to high (10) (scale shown only to 5). Flavor notes are abbreviated as follows; Met (metallic), EtOH (alcohol), Iso (iso-amyl acetate), Swe (sweetness), HS (hydrogen sulfide), Oxi (oxidation/papery), Malt (malt character), Auto (autolysis/meaty) 4-Vg (4-vinyl guaiacol), Bi (bitterness), Di (diacetyl), DMS (di-methyl sulfide), Fruit (fruity/esters).

To determine whether it was possible to use killer yeasts to prevent hyperattenuation by diastatic yeasts, two 1,000 L brewing trials were conducted using the non-killer brewing strain WLP-001 (Fig. 4A). Both fermentations proceeded normally in the first 6 days, with some variability in the gravity readings in the first 36-h period due to the rapid evolution of CO_2_ (Table S3). After approximately 100 h of stable readings, fermentations were judged to have reached terminal gravity [~1.6° Plato (P)]. In trial one, the diastatic phenolic off-flavor (POF)+ yeast strain Belle Saison (Lallemand Inc.) was added to a final concentration of 5 × 10^4^ cells mL^−1^. The addition of the diastatic yeast cells resulted in a rapid drop in gravity to 1.06° P (Fig. 4B) as well as an increase in pH (Fig. 4C) and temperature (Fig. 4D) before the trial was halted. This indicated that diastatic yeasts could ferment saccharides derived from the hydrolysis of residual starches and dextrins in the finished beer. For trial two, as the diastatic yeast Belle Saison was sensitive to the K2 killer toxin, remediation of a simulated contamination event was trialed by adding the K2 killer yeast strain Viva (Renaissance Yeast) that was chosen because of its routine use in the brewing industry (Fig. 1). Moreover, Viva is a POF− strain with suitable alcohol tolerance, desirable ester profile, and reduced production of hydrogen sulfide and 4-vinyl guaiacol. Many of the characteristics of Viva are shared with the primary brewing strain WLP-001. The diastatic and killer yeast strains were added simultaneously to a final concentration of 5 × 10^4^ cells mL^−1^. In contrast to trial one, the gravity in trial two dropped by only 0.08° P before recovering to 1.80° P at the end of the trial (Fig. 4B). The pH (Fig. 4C) and temperature (Fig. 4D) remained stable.

To assess the effect of diastatic remediation on flavor profile, a sensory panel of trained cicerones performed a hedonic rating like/dislike and off-flavor evaluation on a 10-point scale. The yeast strain used in these trials (WLP-001) is commonly used in brewing and is characterized by a clean and fruity aroma (Fig. 4E). While it was evident that adding a K2 killer yeast prevented hyperattenuation, there was still a noticeable and undesirable flavor to the final brew. Specifically, while the beer produced from both diastatic trials maintained several desirable flavor characteristics (fruity/sweet/malty), they were very expressive of 4-vinyl guaiacol (4-Vg), which presented as clove or allspice, with a sensory score of 5 out of 10 in both fermentation trials (Fig. 4F). This off-flavor was present with or without adding the POF− K2 killer yeast strain (Viva) despite preventing hyperattenuation. In addition, the trial with the K2 killer yeast had notes of an autolysis/meaty flavor (Auto) that we attribute to the successful killing and lysis of the diastatic strain in this trial by the K2 killer toxin.

## DISCUSSION

For decades, killer toxins have been proposed as an alternative to synthetic and inorganic fungicides to control pathogenic and spoilage fungi. However, their narrow spectrum of antifungal activity and general instability has likely limited the application of killer yeast. The evolution of the *STA1* gene is a unique genome innovation present in two clades of *S. cerevisiae,* one clade includes brewing yeasts while the other includes yeasts isolated from humans (5, 6). Given the low genetic diversity of diastatic yeasts, these clades have similar killer toxin susceptibilities, as killer toxin sensitivity can be related to phylogenetic distance for some species (47, 48). This would suggest a unique opportunity for the application of killer toxins as an approach to prevent diastatic contamination in craft breweries.

Several strains of diastatic yeasts are resistant to canonical K1 and K2 toxins. For K2, this resistance was due to the acquisition of totiviruses and M2 satellite dsRNAs that provided preprotoxin-mediated immunity essential for the self-protection of killer yeasts from their toxins (15, 54). Although K1 immunity can also be linked to preprotoxin immunity, K1 resistance in diastatic yeasts was independent of satellite dsRNAs and likely due to unknown genome-encoded immunity determinants. Prior large-scale screens of genome deletion libraries have demonstrated that many cellular pathways can contribute to killer toxin immunity, and *S. cerevisiae* can rapidly evolve K1-resistance in cell culture (42, 55–59). A recent genome-wide association study of K28 resistance in *S. cerevisiae* identified polymorphic alleles of *KTD1* that dictated K28 susceptibility (60). Similarly, truncated killer toxin genes analogous to the minimal preprotoxin immunity domain of K1 have been found in the genomes of several species of Saccharomycotina yeasts (41). The acquisition of dsRNA satellites and the presence of anti-toxin defenses in yeasts suggest that the application of killer toxins in craft breweries could drive the evolution of killer toxin resistance in diastatic yeasts. However, the prevalence and diversity of killer toxins in *S. cerevisiae* motivate the screening for killer toxins that would overcome evolved resistance in diastatic yeasts (32, 33). The mutations identified in K2v broaden the spectrum of antifungal activity against diastatic yeasts compared to canonical K2. Previous studies have identified a variant K2 toxin named K3 based on differences in the spectrum of activity and dsRNA satellite size (61, 62). Similarly, polymorphisms in K1 have also been shown to alter the potency and antifungal specificity of K1 (33, 63). Therefore, a better understanding of how mutations improve the efficacy of killer toxins will benefit their future application against diastatic yeasts and other pathogens and spoilage fungi.

Proof-of-concept fermentation trials show that killer toxins effectively prevent diastatic hyperattenuation resulting from the growth of *STA1+ Saccharomyces cerevisiae*. Similar protection has been observed in the winemaking industry, where killer yeasts are widely used and can prevent contamination by undesirable strains of *Saccharomyces* but not non-*Saccharomyces* species of yeasts (64). In situations where a brewery actively monitors for the presence of diastatic yeasts during fermentation, the addition of killer yeasts or enriched killer toxins to a contaminated fermenter could be an approach to prevent product loss. However, the success of remediation would likely depend on the extent of diastatic contamination, and there is a need to define the number of killer yeasts or concentration of toxin required to prevent hyperattenuation. Future experiments to investigate the population dynamics after killer yeast or toxin remediation would yield valuable insights into the viability of diastatic yeast cells after treatment and the long-term stability of yeast toxins in beer.

In the fermentation trials to remediate diastatic contamination using K2 killer yeasts, the beer produced had a noticeable and undesirable phenolic flavor despite preventing hyperattenuation. The brewing strain WLP-001 was used in the primary fermentation for its clean aroma profile and POF− status; thus, the phenolic flavor after diastatic contamination was attributed to the addition of the diastatic POF+ Belle Saison yeast used in the trial. In these trials, a high final concentration of diastatic yeast was added (5 × 10^4^ cells mL^−1^), considerably higher than the threshold for contamination in the brewing process (13). Therefore, the large bolus of diastatic yeasts was expected to be responsible for the undesirable flavor characteristics of the beer produced by these trials. Under more realistic scenarios with lower numbers of diastatic yeasts invading the brewing process, lower concentrations of the killer toxin in beer would likely be sufficient to prevent hyperattenuation and undesirable flavors. Indeed, killer toxins can trigger the cell death of susceptible yeasts at lower concentrations than those required for cell lysis (65, 66).

As many craft breweries do not actively monitor for diastatic contamination, an alternative approach to safeguard against contamination could be engineering brewing strains to produce killer toxins during fermentation. Killer toxin genes could be introduced into the yeast genome by selective breeding or direct genome editing, as has been demonstrated for winemaking yeasts (67–69). Alternatively, totiviruses and satellite dsRNAs that encode killer toxins could be introduced into existing brewing strains by cytoduction (70). Engineered brewing yeasts have solved many fermentation-related problems for craft brewers (71). Practical examples include lactic acid-producing yeast, diacetyl-free yeasts expressing alpha acetolactate decarboxylase, and yeasts expressing β-lyase to produce aromatic thiols. These yeasts allow for much faster fermentation times and save brewers money in labor and materials while enhancing the taste and flavor of the beer. As yeasts are pitched into wort at high densities, killer toxin concentrations are predicted to increase rapidly during fermentation. Thus, killer toxins in wort could prevent the invasion of diastatic yeasts into the brewing process at any downstream production stage.

*S. cerevisiae* acidifies wort during fermentation to a pH of ~4.2, which is optimal for killer toxin activity (72, 73). The stability of killer toxins in the finished beer remains to be investigated, but it is conceivable that at low pH and ambient temperatures, killer toxins would remain active during the packaging process and protect against diastatic contamination. Alternatively, killer yeast could also be used for “conditioning,” whereby yeast is added during packaging for natural carbonation. This would allow for killer toxin production in the packaged beer, protecting the finished product from diastatic yeast invasion. Regardless of the method of killer yeast application in craft breweries, the most crucial consideration would be to ensure desirable fermentation profiles, flavor, and shelf life. Therefore, developing killer brewing strains will be a priority to realize the successful industrial application of killer toxins.

## MATERIALS AND METHODS

### Microbial strains and growth conditions

The names and origins of strains used in this study are listed in Table 1. Yeasts were propagated in standard yeast extract, peptone, and dextrose medium (YPD; 10 g yeast extract, 20 g peptone, 20 g dextrose, and 20 g agar in a total volume of 1L of deionized water). Yeast strains containing killer toxin expression plasmids were maintained using complete medium (CM; 2.5 g appropriate amino acid mixture, 1.7 g yeast nitrogen base, 5 g ammonium sulfate, 20 g dextrose, and 20 g agar in a total volume of 1L of deionized water) lacking uracil. *Escherichia coli* for cloning was grown using Luria broth (LB) with an appropriate antibiotic and agar (LB; 25 g LB powder, 15 g agar with a final concentration of either 10 µg mL^−1^ of spectinomycin or 100 µg mL^−1^ of ampicillin).

**TABLE 1:** Yeast strains used in the current study^*a*^

Genus	Species	Strain	Brewing nomenclature	STA1	Killer	POF	Source
*Saccharomyces*	*cerevisiae*	OYL-019	Belgian Ale D yeast	+	nd^*b*^	+	Omega Brewing
*Saccharomyces*	*cerevisiae*	OYL-025	Bavarian Wheat I yeast	+	-	+	Omega Brewing
*Saccharomyces*	*cerevisiae*	OYL-026	French Saison yeast	+	nd	+	Omega Brewing
*Saccharomyces*	*cerevisiae*	OYL-027	Belgian Saison yeast	+	nd	+	Omega Brewing
*Saccharomyces*	*cerevisiae*	OYL-039	Biere de garde yeast	+	nd	+	Omega Brewing
*Saccharomyces*	*cerevisiae*	OYL-040	Belgian Dark Ale	+	nd	+	Omega Brewing
*Saccharomyces*	*cerevisiae*	OYL-042	Belgian Saison II yeast	+	nd	+	Omega Brewing
*Saccharomyces*	*cerevisiae*	OYL-055	Vermont Farmhouse Ale	+	nd	+	Omega Brewing
*Saccharomyces*	*cerevisiae*	OYL-056	Belgian golden strong yeast	+	-	+	Omega Brewing
*Saccharomyces*	*cerevisiae*	OYL-112	Swiss Lager	+	-	+	Omega Brewing
*Saccharomyces*	*cerevisiae*	OYL-200	Tropical IPA yeast	+	nd	-	Omega Brewing
*Saccharomyces*	*cerevisiae*	OYL-205	Tropical IPA	+	nd	-	Omega Brewing
*Saccharomyces*	*cerevisiae*	OYL-500	Saisonstein yeast	+	nd	+	Omega Brewing
*Saccharomyces*	*cerevisiae*	OYL-501	Gulo yeast	+	nd	-	Omega Brewing
*Saccharomyces*	*cerevisiae*	TVM	STA1	+	nd	nd	Omega Brewing
*Saccharomyces*	*cerevisiae*	Belle Saison	French Saison yeast	+	nd	+	Lallamand
*Saccharomyces*	*cerevisiae*	AAB	N/A^*c*^	+	nd	nd	Gianni Liti
*Saccharomyces*	*cerevisiae*	AAQ	N/A	+	nd	nd	Gianni Liti
*Saccharomyces*	*cerevisiae*	AAR	N/A	+	nd	nd	Gianni Liti
*Saccharomyces*	*cerevisiae*	AEQ	N/A	+	nd	nd	Gianni Liti
*Saccharomyces*	*cerevisiae*	AFA	N/A	+	-	nd	Gianni Liti
*Saccharomyces*	*cerevisiae*	AFB	N/A	+	+	nd	Gianni Liti
*Saccharomyces*	*cerevisiae*	AFP	N/A	+	-	nd	Gianni Liti
*Saccharomyces*	*cerevisiae*	APP	N/A	+	+	nd	Gianni Liti
*Saccharomyces*	*cerevisiae*	AQG	N/A	+	nd	nd	Gianni Liti
*Saccharomyces*	*cerevisiae*	AQH	N/A	+	+	nd	Gianni Liti
*Saccharomyces*	*cerevisiae*	ASB	N/A	+	-	nd	Gianni Liti
*Saccharomyces*	*cerevisiae*	BRM	N/A	+	-	nd	Gianni Liti
*Saccharomyces*	*cerevisiae*	CBN	N/A	+	nd	nd	Gianni Liti
*Saccharomyces*	*cerevisiae*	CFF	N/A	+	nd	nd	Gianni Liti
*Saccharomyces*	*cerevisiae*	CPD	N/A	+	nd	nd	Gianni Liti
*Saccharomyces*	*cerevisiae*	SACE_YAB	N/A	+	-	nd	Gianni Liti
*Saccharomyces*	*cerevisiae*	SACE_YAG	N/A	+	-	nd	Gianni Liti
*Saccharomyces*	*cerevisiae*	SACE_YDD	N/A	+	-	nd	Gianni Liti
*Saccharomyces*	*cerevisiae*	VIC-23	Viva	-	+ [M2]	-	Renaissance Yeast
*Saccharomyces*	*cerevisiae*	WLP-001	California Ale Yeast	-	-	-	White Labs
*Saccharomyces*	*cerevisiae*	BY4741	N/A	nd	-	nd	n/a
*Saccharomyces*	*cerevisiae*	CHB	N/A	nd	+ [M2v]	nd	Gianni Liti
*Saccharomyces*	*cerevisiae*	SACE_YCA	N/A	nd	+ [M2v]	nd	Gianni Liti
*Saccharomyces*	*cerevisiae*	BLG	N/A	nd	+ [M2v]	nd	Gianni Liti
*Saccharomyces*	*cerevisiae*	ACP	N/A	nd	+ [M2v]	nd	Gianni Liti
*Saccharomyces*	*cerevisiae*	CHD	N/A	nd	+ [M2v]	nd	Gianni Liti
*Saccharomyces*	*cerevisiae*	BSG	N/A	nd	+ [M2v]	nd	Gianni Liti
*Saccharomyces*	*cerevisiae*	CYC1058	N/A	nd	+ [M2]	nd	CYC
*Saccharomyces*	*cerevisiae*	CYC1172	N/A	nd	+ [M2]	nd	CYC
*Saccharomyces*	*cerevisiae*	DMS 70454	N/A	nd	+ [Mlus]	nd	DSMZ
*Saccharomyces*	*cerevisiae*	YSM1307	N/A	nd	+ [M1]	nd	FGSC
*Saccharomyces*	*cerevisiae*	OS179	N/A	nd	+ [M62]	nd	Gianni Liti
*Saccharomyces*	*cerevisiae*	OS40	N/A	nd	+ [M21]	nd	Gianni Liti
*Saccharomyces*	*cerevisiae*	MS300C	N/A	nd	+ [M28]	nd	Manfred Schmitt
*Saccharomyces*	*cerevisiae*	OS294	N/A	nd	+ [M74]	N/A	Gianni Liti
*Saccharomyces*	*paradoxus*	Y63717	N/A	nd	+ [M1L]	nd	FGSC
*Naumovozyma*	*dairenensis*	NCYC777	N/A	nd	+	nd	NCYC

^*a*^ '+' and '-' are binary measures of the presence of a specific gene (*STA1*), the killer phenotype (killer), or POF.

^*b*^ nd, not done.

^*c*^ N/A, not applicable.

### TOPO and gateway cloning of K1, K2, and K2v

To amplify the full-length K1, K2 and K2v genes, SuperScript IV reverse transcriptase (18090010; Thermo) and Phusion DNA Polymerase (M30530S; New England Biolabs) were used with the primer pairs PRX542/PRUI1, PRUI115/PRUI116, and K2P1/K2P2, respectively (Table 2). The templates for these reactions were purified dsRNAs from *S. cerevisiae* strains YJM1307 (K1), ACP (K2v), and CYC1172 (K2). After cleanup with the QIAquick PCR purification kit, A-tails were added to the PCR products using Taq polymerase (M0273S; New England Biolabs) following the manufacturer’s recommendation. A-tailed PCR products were cloned using the pCR8/GW/TOPO TA Cloning Kit by mixing 0.25 µL of salt solution and 0.25 µL of pCR8 vector with 1 µL of the PCR product. The solution was incubated at 25°C for 1.5 h. Half of the manufacturer’s recommended amount of One Shot TOP10 chemically competent *E. coli* (25 µL) was added to the reaction mix on ice. The mixture was then incubated on ice for 30 min, followed by 30 s at 42°C and 2 min on ice. 250 µL of pre-warmed (37°C) SOC medium was added, and the mixture was shaken at 37°C at 220 rpm for 1 h before being spread on LB agar plates containing spectinomycin. Plasmids were purified using the QIAprep Spin Miniprep Kit before analysis by restriction enzyme analysis and Sanger sequencing (using primers M13F and M13R). The insertion of K1 (YJM1307), K2v (ACP), and K2 (CYC1172) into pCR8/GW/TOPO created the plasmids pUI101, pVZ001, and pUI099. Gateway cloning introduced K1, K2, and K2v genes into an integrative yeast shuttle vector (pAG306-GPD-ccdB). One-quarter of the manufacturer’s recommended amount of each reagent was used for each reaction: 0.5 µL of the pCR8 entry vector, 0.5 µL LR Clonase II enzyme mix, 0.5 µL destination plasmid, and 1 µL of sterile water (74). The mixture was inoculated at 25°C for 3 h before adding 0.25 µL of Proteinase K and incubating at 37°C for 10 min. Reaction mixtures were used to transform One Shot TOP10 chemically competent *E. coli* as described above but with selection by ampicillin. Plasmids were purified using the QIAprep Spin Miniprep Kit before being analyzed using restriction enzyme analysis. The Gateway cloning of K2 (CYC1172) and K2v (ACP) into the high copy vector pAG426-GPD-ccdB created the plasmids pUI095 and pVZ004, respectively. The Gateway cloning of K1 (YJM1307) and K2 (CYC1172) into the integrative vector pAG306-GPD-ccdB created the plasmids pVZ002 and pVZ003, respectively (Table 3). All plasmid sequences are in File S1.

**TABLE 2:** DNA primers used in the current study

Name	Nucleotide sequence	Target
prMRK199	TGTCGGCTAATGGTAACCTGTATGG	K1 gene
prMRK120	GTCACAGCCTTCAAAGTCATTATTGG	K1 gene
prMRK123	GTGGCCTCTTTTTATTCACCACTCC	K2 gene
prMRK124	GTCTCGAATCCCTCTTGACAATTCC	K2 gene
K2P1	ATGAAAGAGACTACCACCAGC	K2v gene
K2P2	GATCGGCGACAGTGTAAGTGGT	K2v gene
PRUI115	ATGAAAGAGACTACCACCAGCCTGATGC	K2 gene
PRUI116	CTAGCCGCTGTCACATTCACCATCAACC	K2 gene
PRX542	GAAAAATAAAGAAATGACGAAGCCAACCCAAG	K1 gene
PRUI1	GAGTTATCGCATCAGAGGTCAGACAC	K1 gene

**TABLE 3:** DNA plasmids used in the current study

Name	Description	Yeast marker	Bacterial marker	Reference
pAG306-GPD-ccdB	Gateway destination vector; integrative shuttle vector	*URA3*	*bla, cat*	(74)
pCR8/GW/TOPO	TOPO-TA cloning vector and Gateway entry vector	n/a^*a*^	*aad*	Thermofisher
pAG426-GPD-ccdB	Gateway destination vector; high copy episomal shuttle vector	*URA3*	*bla, cat*	(74)
pUI099	pCR8 with K2 (CYC1172)	n/a	*aad*	This study
pVZ001	pCR8 with K2v (ACP)	n/a	*aad*	This study
pUI101	pCR8 with K1 (YJM1307)	n/a	*aad*	This study
pUI095	pAG426-GPD-ccdB with K2	*URA3*	*bla*	This study
pVZ004	pAG426-GPD-ccdB with K2v	*URA3*	*bla*	This study
pVZ002	pAG306-GPD-ccdB with K1	*URA3*	*bla*	This study
pVZ003	pAG306-GPD-ccdB with K2	*URA3*	*bla*	This study

^*a*^ n/a, not applicable.

### Curing of satellite dsRNAs

Yeast strains to be cured of satellites were cultured in 25 mL of YPD media at 30°C with shaking at 180 RPM to OD 1. 1 mL of this culture was added to 3 mL of YPD with increasing concentration of cycloheximide (1 μM–14 µM). Cells were incubated for ~5 days at 30°C at 180 RPM. 100 µL of these liquid cultures was spread over 10 cm YPD agar plates and incubated for 48 h at 30°C. The resulting colonies were then examined for loss of killer toxin production.

### Double-stranded RNA extraction

Double-stranded RNAs for analysis by gel electrophoresis were purified according to the method described by Fredericks et al. (41). Specifically, yeast cultures inoculated in YPD broth were grown overnight at 30°C. Cultures were centrifuged for 5 min at 8,000 × *g*, the supernatant was aspirated, and the cells were washed once with sterile water. Cellulose columns were prepared by puncturing a 0.6 mL tube with a hot needle and nesting it in a 2.0 mL centrifuge tube. 0.06 g of cellulose powder D (Advantec, Japan) was added to the 0.6 mL tube, followed by 500 µL of wash buffer [1 × STE (100 mM NaCl; 10 mM Tris–HCl, pH 8.0; 1 mM EDTA, pH 8.0) containing 16% (vol/vol) ethanol]. 1 × STE was added to approximately 0.04 g of wet biomass from YPD cultures and was vortexed for 3 min at 3,000 rpm (Disruptor Genie, Scientific Industries, Bohemia, NY, USA). 50 µL of 10% (wt/vol) SDS solution and 500 µL of phenol–chloroform–isoamyl alcohol (25:24:1) pH 8.0 were added to the cell suspension and vortexed until homogeneous. Samples were centrifuged at 20,000 × *g* for 5 min, the supernatant was transferred to a clean tube, and a second 500 µL of phenol–chloroform–isoamyl alcohol extraction was performed. The aqueous phase was transferred to a clean tube, and a one-fifth volume of ethanol was added. Tubes were mixed and centrifuged at 20,000 × *g* for 3 min before the supernatant was transferred to the cellulose column and centrifuged at 10,000 × *g* for 10 s. After discarding the flow-through, 400 µL of wash buffer was added to the columns, centrifuged at 10,000 × *g* for 10 s, and the flow-through was discarded three times. The columns were dried by centrifugation at 10,000 × *g* for 10 s. Cellulose columns were transferred to clean tubes, 400 µL of 1 × STE was added, and columns were centrifuged at 10,000 × *g* for 10 s to collect the eluate. 40 µL of 3 M aqueous sodium acetate (pH 5.2) and 1 mL of 100% ethanol were added to the eluate, mixed by inversion, and centrifuged at 20,000 × *g* for 5 min to precipitate the dsRNAs. DsRNA pellets were allowed to air-dry before being suspended in nuclease-free water.

### Killer toxin assays on agar plates

The general detection of killer toxin production by killer yeasts was assayed as previously described by Fredericks et al. (41) by growing yeasts on killer yeast agar plates (YPD agar plates with 0.003% wt/vol methylene blue buffered at pH 4.6 with sodium citrate). General screening for toxin production was done by observing either a zone of growth inhibition and/or methylene blue-staining of the susceptible lawn yeasts. Specifically, approximately 6 × 10^5^ toxin-susceptible yeast cells were spread onto killer yeast agar plates. Cells from 2 mL overnight cultures of killer yeasts were pelleted and pinned onto susceptible lawns for qualitative assessment of killer toxin susceptibility. Approximately 6 × 10^6^ cells of killer toxin-expressing yeast were spotted on the inoculated plates for quantitative assays. Killer assay plates were incubated at ambient temperature for 3–7 days. Killer toxin growth inhibition measurements were made after 7 days of growth using images taken by a Singer Instruments Phenobooth + colony counter. The images were analyzed in Fiji (Image J), where the colony’s diameter and the inhibition area were measured. When killer toxin expression from high copy plasmids [K2v (pUI095) and K2 (pVZ004)] was induced by galactose. These strains of yeasts were maintained on CM lacking uracil before plating onto YPD or YPG plates (yeast peptone galactose) plates.

### Detection of K1 and K2 killer toxin genes

The presence of killer toxin genes on satellite dsRNAs was detected in total nucleic acid samples [prepared according to reference (75)] using SuperScript IV reverse transcriptase (Thermo) and Phusion DNA Polymerase (New England Biolabs) as directed by the manufacturer’s instructions. Primer pairs prMRK199/prMRK120 and prMRK123/prMRK124 were used to detect the canonical killer toxin genes K1 and K2, respectively. For the detection of these genes on DNA, reverse transcription was omitted. The PCR products were visualized using 1% agarose gel at 120V for 45 min. All primers are listed in Table 2. Genomic DNA was extracted from *S. cerevisiae* strains with the integrated plasmids pVZ002 and pVZ003 encoding K1 and K2, respectively.

### Fermentation trials

All yeasts were grown from pure cultures maintained cryogenically or on agar slants. Brewing yeasts for this project were propagated in four parallel flasks utilizing a stepwise 10-fold increase in volume at each step. This process started with inoculation from an agar slant into 25 mL of sterile 12°P wort into two 125 mL baffled culture flasks and placed into a shaker incubator at 28°C. This was repeated to a final volume of 5 L and a final cell count of 5.0 × 10^8^ cells per ml. Fermentation trials were brewed with Rahr two-row brewers’ malt and 363 g of bravo hops (20 IBU). The 11.5°P wort was transferred out of the brewhouse through an inline heat exchanger to reduce wort temperature to 20°C and inoculated with 10 L of WLP-001 at a pitching rate of 1.0 × 10^6^ cell/ml/°P. For both the diastatic and killer yeasts, 5 × 10^4^ cells mL^−1^ were added once the gravity had stabilized for three consecutive days. The temperature during fermentation was maintained at 21°C. In trial one, 5 L of the *STA1+* diastatic yeast strain Belle Saison from Lallemand was added through the hop port while CO_2_ provided positive pressure. In trial two, 5 L of the same diastatic was added with 5 L of the K2 killer yeast strain Viva (VIC-23) from Renaissance Yeast through the hop port while CO_2_ provided positive pressure. Cell counts were taken using an AOPI stain on the Nexcelom X2 automated cellometer. All data from both trials were collected in real time *via* a recirculating inline loop attached *via* the hop port. The instrument collected data every 30 min on pH, density (g/cm^3^), gravity (°P), dissolved oxygen (DO) (mg/L), conductivity (uS/cm), and temperature (°C) using the Brew IQ real-time data collection system. The instrumentation was cleaned with the alkaline non-caustic CIP cleaner Cell-R-Mastr, triple rinsed with 60°C water, and sanitized with peroxyacetic acid for 30 min before attaching to the fermenter.

### Fermentation cellar cooling systems

This study’s 10-hectoliter (1,000 L) pioneer fermentation vessels were jacketed and cooled *via* an inline re-circulating propylene glycol system. This closed-loop system employed a heat transfer fluid of propylene glycol and water that circulates to the fermenter jackets through heat exchangers. A 30RAP011 Carrier 10 Ton Glycol Chilling Unit was used to cool the 50% glycol-water mixture to 21°C and was monitored and controlled by an Allen Bradley human-machine interface.

### Tasting panel

A panel of five Cicerones and trained tasters from Rhinegeist was selected to participate in the off-flavor evaluation of the beer samples from both fermentation trials (males, aged 34, 33, 35, 38, and 39). Cicerones are rigorously trained to refine their olfactory and senses, enabling them to detect and differentiate specific off-flavors commonly associated with beer. Triad panels in parallel were conducted blind to determine whether tasters could detect any difference between the two samples. Each beer was sampled in 118 mL pours in clear snifter glasses. Tasters were asked to rate the intensity of the selected aromas and tastes in each sample on a scale from 0 to 10, with 0 being absent and 10 being high. The tasting panel was conducted in accordance with the ethical standards of the Institutional Review Board (IRB) of the University of Idaho (Protocol Number: 24–040, Reference: 028450) and was judged to be exempt from human subjects research.

## SUPPLEMENTAL MATERIAL

File S1 aem.01072-24-s0001.txt Plasmid sequences.

Figure S1 aem.01072-24-s0002.tif K2 killer toxin production by diastatic yeasts causes K2 resistance.

Figure S2 aem.01072-24-s0003.tif K2 killer toxin production by diastatic yeasts causes K2 resistance.

Table S1 aem.01072-24-s0004.csv Susceptibility of diastatic yeasts to canonical killer toxins.

Table S2 aem.01072-24-s0005.csv Killer yeasts that inhibit K1- and K2-resistant strains of diastatic yeasts.

Table S3 aem.01072-24-s0006.csv Brewing trial data.

ASM does not own the copyrights to Supplemental Material that may be linked to, or accessed through, an article. The authors have granted ASM a non-exclusive, world-wide license to publish the Supplemental Material files. Please contact the corresponding author directly for reuse.

## References

[1] Baiano A. 2021. Craft beer: an overview. Comp Rev Food Sci Food Safe 20:1829–1856. doi:10.1111/1541-4337.12693 33369039

[2] Worrell E, Galitsky C, Martin N. 2006. Energy efficiency opportunities in the brewery industry

[3] Tokos H, Novak Pintarič Z. 2009. Synthesis of batch water network for a brewery plant. J Clean Prod 17:1465–1479. doi:10.1016/j.jclepro.2009.06.009

[4] Kaneda H, Kano Y, Osawa T, Kawakishi S, Koshino S. 1994. Free radical reactions in beer during pasteurization. Int J of Food Sci Tech 29:195–200. doi:10.1111/j.1365-2621.1994.tb02061.x

[5] Peter J, De Chiara M, Friedrich A, Yue J-X, Pflieger D, Bergström A, Sigwalt A, Barre B, Freel K, Llored A, Cruaud C, Labadie K, Aury J-M, Istace B, Lebrigand K, Barbry P, Engelen S, Lemainque A, Wincker P, Liti G, Schacherer J. 2018. Genome evolution across 1,011 Saccharomyces cerevisiae isolates. Nature 556:339–344. doi:10.1038/s41586-018-0030-5 29643504 PMC6784862

[6] Krogerus K, Gibson B. 2020. A re-evaluation of diastatic Saccharomyces cerevisiae strains and their role in brewing. Appl Microbiol Biotechnol 104:3745–3756. doi:10.1007/s00253-020-10531-0 32170387 PMC7162825

[7] Tamaki H. 1978. Genetic studies of ability to ferment starch in Saccharomyces: gene polymorphism. Molec Gen Genet 164:205–209. doi:10.1007/BF00267385

[8] Yamashita I, Maemura T, Hatano T, Fukui S. 1985. Polymorphic extracellular glucoamylase genes and their evolutionary origin in the yeast Saccharomyces diastaticus. J Bacteriol 161:574–582. doi:10.1128/jb.161.2.574-582.1985 3918018 PMC214921

[9] Pretorius IS, Marmur J. 1988. Localization of yeast glucoamylase genes by PFGE and OFAGE. Curr Genet 14:9–13. doi:10.1007/BF00405847 3138030

[10] Begrow W. 2017. Fighting quality threats: notable microbiological contaminations of craft beer in the United States. Brewing and Beverage Industry International

[11] M-Dörnberg T, Jacob F, Hutzler M. 2017. Incidence of Saccharomyces cerevisiae var. diastaticus in the beverage Industry: cases of contamination, 2008–2017. Mast Brew Assoc Am Tech Quart 54:140–148. doi:10.1094/TQ-54-4-1130-01

[12] Latorre M, Hutzler M, Michel M, Zarnkow M, Jacob F, Libkind D. 2020. Genotypic diversity of Saccharomyces cerevisiae spoilers in a community of craft microbreweries. Brew Sci 73:51–57. doi:10.23763/BrSc20-06latorre

[13] Burns LT, Sislak CD, Gibbon NL, Saylor NR, Seymour MR, Shaner LM, Gibney PA. 2021. Improved functional assays and risk assessment for STA1+ strains of Saccharomyces cerevisiae. J Am Soc Brew Chem 79:167–180. doi:10.1080/03610470.2020.1796175

[14] Navarro Y, Torija M-J, Mas A, Beltran G. 2020. Viability-PCR allows monitoring yeast population dynamics in mixed fermentations including viable but non-culturable yeasts. Foods 9:1373. doi:10.3390/foods9101373 32992467 PMC7600988

[15] Schmitt MJ, Breinig F. 2006. Yeast viral killer toxins: lethality and self-protection. Nat Rev Microbiol 4:212–221. doi:10.1038/nrmicro1347 16489348

[16] Schaffrath R, Meinhardt F, Klassen R. 2018. Yeast killer toxins: fundamentals and applications, p 87–118. In T, A, A, S (ed), Physiology and genetics. The mycota (A comprehensive treatise on fungi as experimental systems for basic and applied research). Springer, Cham.

[17] Marquina D, Santos A, Peinado JM. 2002. Biology of killer yeasts. Int Microbiol 5:65–71. doi:10.1007/s10123-002-0066-z 12180782

[18] Boynton PJ, Greig D. 2014. The ecology and evolution of non-domesticated Saccharomyces species. Yeast 31:449–462. doi:10.1002/yea.3040 25242436 PMC4282469

[19] Jijakli M, de CD, Dickburt C, Lepoivre P. 2002. Pre-and post-harvest practical application of Pichia anomala strain K, beta-1, 3-glucans and calcium chloride on apples: two years of monitoring and efficacy against post-harvest diseases. JOBC wprs Bulletin 25:29–32.

[20] Kitamoto HK, Ohmomo S, Nakahara T. 1993. Selection of killer yeasts (Kluyveromyces lactis) to prevent aerobic deterioration in silage making. J Dairy Sci 76:803–811. doi:10.3168/jds.S0022-0302(93)77404-4 8463490

[21] Liu S-Q, Tsao M. 2009. Inhibition of spoilage yeasts in cheese by killer yeast Williopsis saturnus var. saturnus. Int J Food Microbiol 131:280–282. doi:10.1016/j.ijfoodmicro.2009.03.009 19349088

[22] Lowes KF, Shearman CA, Payne J, MacKenzie D, Archer DB, Merry RJ, Gasson MJ. 2000. Prevention of yeast spoilage in feed and food by the yeast mycocin HMK. Appl Environ Microbiol 66:1066–1076. doi:10.1128/AEM.66.3.1066-1076.2000 10698773 PMC91944

[23] Platania C, Restuccia C, Muccilli S, Cirvilleri G. 2012. Efficacy of killer yeasts in the biological control of Penicillium digitatum on Tarocco orange fruits (Citrus sinensis). Food Microbiol 30:219–225. doi:10.1016/j.fm.2011.12.010 22265304

[24] Santos A, Marquina D. 2004. Killer toxin of Pichia membranifaciens and its possible use as a biocontrol agent against grey mould disease of grapevine. Microbiology (Reading) 150:2527–2534. doi:10.1099/mic.0.27071-0 15289549

[25] Perez MF, Contreras L, Garnica NM, Fernández-Zenoff MV, Farías ME, Sepulveda M, Ramallo J, Dib JR. 2016. Native killer yeasts as biocontrol agents of postharvest fungal diseases in lemons. PLoS One 11:e0165590. doi:10.1371/journal.pone.0165590 27792761 PMC5085023

[26] Díaz MA, Pereyra MM, Santander FFS, Perez MF, Córdoba JM, Alhussein M, Karlovsky P, Dib JR. 2020. Protection of citrus fruits from postharvest infection with Penicillium digitatum and degradation of patulin by biocontrol yeast Clavispora lusitaniae 146. Microorganisms 8:1477. doi:10.3390/microorganisms8101477 32993018 PMC7601000

[27] Quijano CD, Wichmann F, Schlaich T, Fammartino A, Huckauf J, Schmidt K, Unger C, Broer I, Sautter C. 2016. KP4 to control Ustilago tritici in wheat: enhanced greenhouse resistance to loose smut and changes in transcript abundance of pathogen related genes in infected KP4 plants. Biotechnol Rep (Amst) 11:90–98. doi:10.1016/j.btre.2016.08.002 28352545 PMC5042339

[28] Schlaich T, Urbaniak BM, Malgras N, Ehler E, Birrer C, Meier L, Sautter C. 2006. Increased field resistance to Tilletia caries provided by a specific antifungal virus gene in genetically engineered wheat. Plant Biotechnol J 4:63–75. doi:10.1111/j.1467-7652.2005.00158.x 17177786

[29] Haïssam JM. 2011. Pichia anomala in biocontrol for apples: 20 years of fundamental research and practical applications. Antonie Van Leeuwenhoek 99:93–105. doi:10.1007/s10482-010-9541-2 21222032

[30] Schnürer J, Jonsson A. 2011. Pichia anomala J121: a 30-year overnight near success biopreservation story. Antonie Van Leeuwenhoek 99:5–12. doi:10.1007/s10482-010-9509-2 20872178

[31] Bevan EA, Makower M. 1963. The physiological basis of the killer character in yeast. In Proc Int Congr Genet:202–203.

[32] Vijayraghavan S, Kozmin SG, Strope PK, Skelly DA, Magwene PM, Dietrich FS, McCusker JH. 2023. RNA viruses, M satellites, chromosomal killer genes, and killer/nonkiller phenotypes in the 100-genomes S. cerevisiae strains. G3 (Bethesda) 13:jkad167. doi:10.1093/g3journal/jkad167 37497616 PMC10542562

[33] Crabtree AM, Taggart NT, Lee MD, Boyer JM, Rowley PA. 2023. The prevalence of killer yeasts and double-stranded RNAs in the budding yeast Saccharomyces cerevisiae. FEMS Yeast Res 23:foad046. doi:10.1093/femsyr/foad046 37935474 PMC10664976

[34] Wickner RB, Fujimura T, Esteban R. 2013. Viruses and prions of Saccharomyces cerevisiae*.* Adv Virus Res 86:1–36. doi:10.1016/B978-0-12-394315-6.00001-5 23498901 PMC4141569

[35] Dignard D, Whiteway M, Germain D, Tessier D, Thomas DY. 1991. Expression in yeast of a cDNA copy of the K2 killer toxin gene. Mol Gen Genet 227:127–136. doi:10.1007/BF00260717 2046653

[36] Schmitt MJ, Tipper DJ. 1995. Sequence of the M28 dsRNA: preprotoxin is processed to an α/β heterodimeric protein toxin. Virology (Auckl) 213:341–351. doi:10.1006/viro.1995.0007 7491759

[37] Rodríguez-Cousiño N, Maqueda M, Ambrona J, Zamora E, Esteban R, Ramírez M. 2011. A new wine Saccharomyces cerevisiae killer toxin (Klus), encoded by a double-stranded RNA virus, with broad antifungal activity is evolutionarily related to a chromosomal host gene. Appl Environ Microbiol 77:1822–1832. doi:10.1128/AEM.02501-10 21239561 PMC3067279

[38] Bostian KA, Elliott Q, Bussey H, Burn V, Smith A, Tipper DJ. 1984. Sequence of the preprotoxin dsRNA gene of type I killer yeast: multiple processing events produce a two-component toxin. Cell 36:741–751. doi:10.1016/0092-8674(84)90354-4 6697395

[39] Rodríguez-Cousiño N, Gómez P, Esteban R. 2017. Variation and distribution of L-A helper totiviruses in Saccharomyces sensu stricto yeasts producing different killer toxins. Toxins (Basel) 9:313–320. doi:10.3390/toxins9100313 29019944 PMC5666360

[40] Rodriguez-Cousiño N, Gómez P, Esteban R. 2022. Expression of the K74 killer toxin from Saccharomyces paradoxus is modulated by the toxin-encoding M74 double-stranded RNA 5' untranslated terminal region. Appl Environ Microbiol 88:e0203021. doi:10.1128/aem.02030-21 35389250 PMC9040610

[41] Fredericks LR, Lee MD, Crabtree AM, Boyer JM, Kizer EA, Taggart NT, Roslund CR, Hunter SS, Kennedy CB, Willmore CG, Tebbe NM, Harris JS, Brocke SN, Rowley PA. 2021. The species-specific acquisition and diversification of a K1-like family of killer toxins in budding yeasts of the Saccharomycotina. PLoS Genet 17:e1009341. doi:10.1371/journal.pgen.1009341 33539346 PMC7888664

[42] Vepštaitė-Monstavičė I, Lukša J, Konovalovas A, Ežerskytė D, Stanevičienė R, Strazdaitė-Žielienė Ž, Serva S, Servienė E. 2018. Saccharomyces paradoxus K66 killer system evidences expanded assortment of helper and satellite viruses. Viruses 10:564. doi:10.3390/v10100564 30332789 PMC6213463

[43] Goto K, Fukuda H, Kichise K, Kitano K, Hara S. 1991. Cloning and nucleotide sequence of the KHS killer gene of Saccharomyces cerevisiae. Agric Biol Chem 55:1953–1958. doi:10.1271/bbb1961.55.1953 1368726

[44] Goto K, Iwatuki Y, Kitano K, Obata T, Kara S. 1990. Cloning and nucleotide sequence of the KHR killer gene of Saccharomyces cerevisiae. Agric Biol Chem 54:979–984. doi:10.1271/bbb1961.54.979 1368554

[45] Martinac B, Zhu H, Kubalski A, Zhou XL, Culbertson M, Bussey H, Kung C. 1990. Yeast K1 killer toxin forms ion channels in sensitive yeast spheroplasts and in artificial liposomes. Proc Natl Acad Sci U S A 87:6228–6232. doi:10.1073/pnas.87.16.6228 1696721 PMC54506

[46] Schmitt MJ, Klavehn P, Wang J, Schönig I, Tipper DJ. 1996. Cell cycle studies on the mode of action of yeast K28 killer toxin. Microbiology (Reading) 142:2655–2662. doi:10.1099/00221287-142-9-2655 8828235

[47] Buzzini P, Turchetti B, Vaughan-Martini AE. 2007. The use of killer sensitivity patterns for biotyping yeast strains: the state of the art, potentialities and limitations. FEMS Yeast Res 7:749–760. doi:10.1111/j.1567-1364.2007.00238.x 17425671

[48] Golubev WI. 1998. Mycocins (Killer toxins), p 1–8. In Kurtzman CP, Fell JW (ed), 4th ed

[49] de Ullivarri MF, Mendoza LM, Raya RR. 2014. Killer activity of Saccharomyces cerevisiae strains: partial characterization and strategies to improve the biocontrol efficacy in winemaking. Antonie Van Leeuwenhoek 106:865–878. doi:10.1007/s10482-014-0256-7 25145824

[50] Middelbeek EJ, Hermans JM, Stumm C, Muytjens HL. 1980. High incidence of sensitivity to yeast killer toxins among Candida and Torulopsis isolates of human origin. Antimicrob Agents Chemother 17:350–354. doi:10.1128/AAC.17.3.350 7191690 PMC283789

[51] Fredericks LR, Lee MD, Eckert HR, Li S, Shipley MA, Roslund CR, Boikov DA, Kizer EA, Sobel JD, Rowley PA. 2021. Vaginal isolates of Candida glabrata are uniquely susceptible to ionophoric killer toxins produced by Saccharomyces cerevisiae. Antimicrob Agents Chemother 65:e0245020. doi:10.1128/AAC.02450-20 33972245 PMC8218651

[52] Walker GM, McLeod AH, Hodgson VJ. 1995. Interactions between killer yeasts and pathogenic fungi. FEMS Microbiol Lett 127:213–222. doi:10.1111/j.1574-6968.1995.tb07476.x 7758935

[53] Santos A, Sánchez A, Marquina D. 2004. Yeasts as biological agents to control Botrytis cinerea. Microbiol Res 159:331–338. doi:10.1016/j.micres.2004.07.001 15646379

[54] Prins RC, Billerbeck S. 2024. The signal peptide of yeast killer toxin K2 confers producer self-protection and allows conversion into a modular toxin-immunity system. Cell Rep 43:114449. doi:10.1016/j.celrep.2024.114449 38985680

[55] Miyamoto M, Furuichi Y, Komiyama T. 2010. Genome-wide screen of Saccharomyces cerevisiae for killer toxin HM-1 resistance 28:27–41. doi:10.1002/yea.1818 20803478

[56] Carroll SY, Stirling PC, Stimpson HEM, Giesselmann E, Schmitt MJ, Drubin DG. 2009. A yeast killer toxin screen provides insights into A/B toxin entry, trafficking, and killing mechanisms. Dev Cell 17:552–560. doi:10.1016/j.devcel.2009.08.006 19853568 PMC2768656

[57] Pagé N, Gérard-Vincent M, Ménard P, Beaulieu M, Azuma M, Dijkgraaf GJP, Li H, Marcoux J, Nguyen T, Dowse T, Sdicu A-M, Bussey H. 2003. A Saccharomyces cerevisiae genome-wide mutant screen for altered sensitivity to K1 killer toxin. Genetics 163:875–894. doi:10.1093/genetics/163.3.875 12663529 PMC1462477

[58] Servienė E, Lukša J, Orentaitė I, Lafontaine DLJ, Urbonavičius J. 2012. Screening the budding yeast genome reveals unique factors affecting K2 toxin susceptibility. PLoS One 7:e50779. doi:10.1371/journal.pone.0050779 23227207 PMC3515549

[59] Buskirk SW, Rokes AB, Lang GI. 2020. Adaptive evolution of nontransitive fitness in yeast. Elife 9:e62238. doi:10.7554/eLife.62238 33372653 PMC7886323

[60] Andreev I, Laidlaw KME, Giovanetti SM, Urtecho G, Shriner D, Bloom JS, MacDonald C, Sadhu MJ. 2023. Discovery of a rapidly evolving yeast defense factor, KTD1 , against the secreted killer toxin K28 . Proc Natl Acad Sci USA 120:e2217194120. doi:10.1073/pnas.2217194120 36800387 PMC9974470

[61] Young TW, Yagiu M. 1978. A comparison of the killer character in different yeasts and its classification. Antonie Van Leeuwenhoek 44:59–77. doi:10.1007/BF00400077 655699

[62] Wingfield BD, Van Der Meer LJ, Pretorius IS, Van Vuuren HJJ. 1990. K3 killer yeast is a mutant K2 killer yeast. Mycol Res 94:901–906. doi:10.1016/S0953-7562(09)81304-X

[63] Crabtree AM, Kizer EA, Hunter SS, Van Leuven JT, New DD, Fagnan MW, Rowley PA. 2019. A rapid method for sequencing double-stranded RNAs purified from yeasts and the identification of a potent K1 killer toxin isolated from Saccharomyces cerevisiae. Viruses 11:70. doi:10.3390/v11010070 30654470 PMC6356530

[64] Gutiérrez AR, Epifanio S, Garijo P, López R, Santamaría P. 2001. Killer yeasts: incidence in the ecology of spontaneous fermentation. Am J Enol Vitic 52:352–356. doi:10.5344/ajev.2001.52.4.352

[65] Sheppard S, Dikicioglu D. 2019. Dynamic modelling of the killing mechanism of action by virus-infected yeasts. J R Soc Interface 16:20190064. doi:10.1098/rsif.2019.0064 30890050 PMC6451399

[66] Reiter J, Herker E, Madeo F, Schmitt MJ. 2005. Viral killer toxins induce caspase-mediated apoptosis in yeast. J Cell Biol 168:353–358. doi:10.1083/jcb.200408071 15668299 PMC2171720

[67] Seki T, Choi E-H, Ryu D. 1985. Construction of killer wine yeast strain. Appl Environ Microbiol 49:1211–1215. doi:10.1128/aem.49.5.1211-1215.1985 16346794 PMC238531

[68] Boone C, Sdicu A-M, Wagner J, Degré R, Sanchez C, Bussey H. 1990. Integration of the yeast K1 killer toxin gene into the genome of marked wine yeasts and its effect on vinification. Am J Enol Vitic 41:37–42. doi:10.5344/ajev.1990.41.1.37

[69] Javadekar VS, SivaRaman H, Gokhale DV. 1995. Industrial yeast strain improvement: construction of a highly flocculent yeast with a killer character by protoplast fusion. J Ind Microbiol 15:94–102. doi:10.1007/BF01569806 7576466

[70] Young TW. 1981. The genetic manipulation of killer character into brewing yeast. J Inst Brew 87:292–295. doi:10.1002/j.2050-0416.1981.tb04039.x

[71] Iattici F, Catallo M, Solieri L. 2020. Designing new yeasts for craft brewing: when natural biodiversity meets biotechnology. Beverages 6:3. doi:10.3390/beverages6010003

[72] Lukša J, Serva S, Servienė E. 2016. Saccharomyces cerevisiae K2 toxin requires acidic environment for unidirectional folding into active state. Mycoscience 57:51–57. doi:10.1016/j.myc.2015.08.003

[73] Pfeiffer P, Radler F. 1984. Comparison of the killer toxin of several yeasts and the purification of a toxin of type K2. Arch Microbiol 137:357–361. doi:10.1007/BF00410734 6375620

[74] Alberti S, Gitler AD, Lindquist S. 2007. A suite of gateway cloning vectors for high-throughput genetic analysis in Saccharomyces cerevisiae. Yeast 24:913–919. doi:10.1002/yea.1502 17583893 PMC2190539

[75] Lõoke M, Kristjuhan K, Kristjuhan A. 2011. Extraction of genomic DNA from yeasts for PCR-based applications. Biotechniques 50:325–328. doi:10.2144/000113672 21548894 PMC3182553

