# Supplementary figures and images for: Inhibition of diastatic yeasts by Saccharomyces killer toxins to prevent hyperattenuation during brewing
Source: Appl Environ Microbiol. 2024 Sep 12;90(10):e01072-24. doi: 10.1128/aem.01072-24 (PMC11497815; doi:10.1128/aem.01072-24)

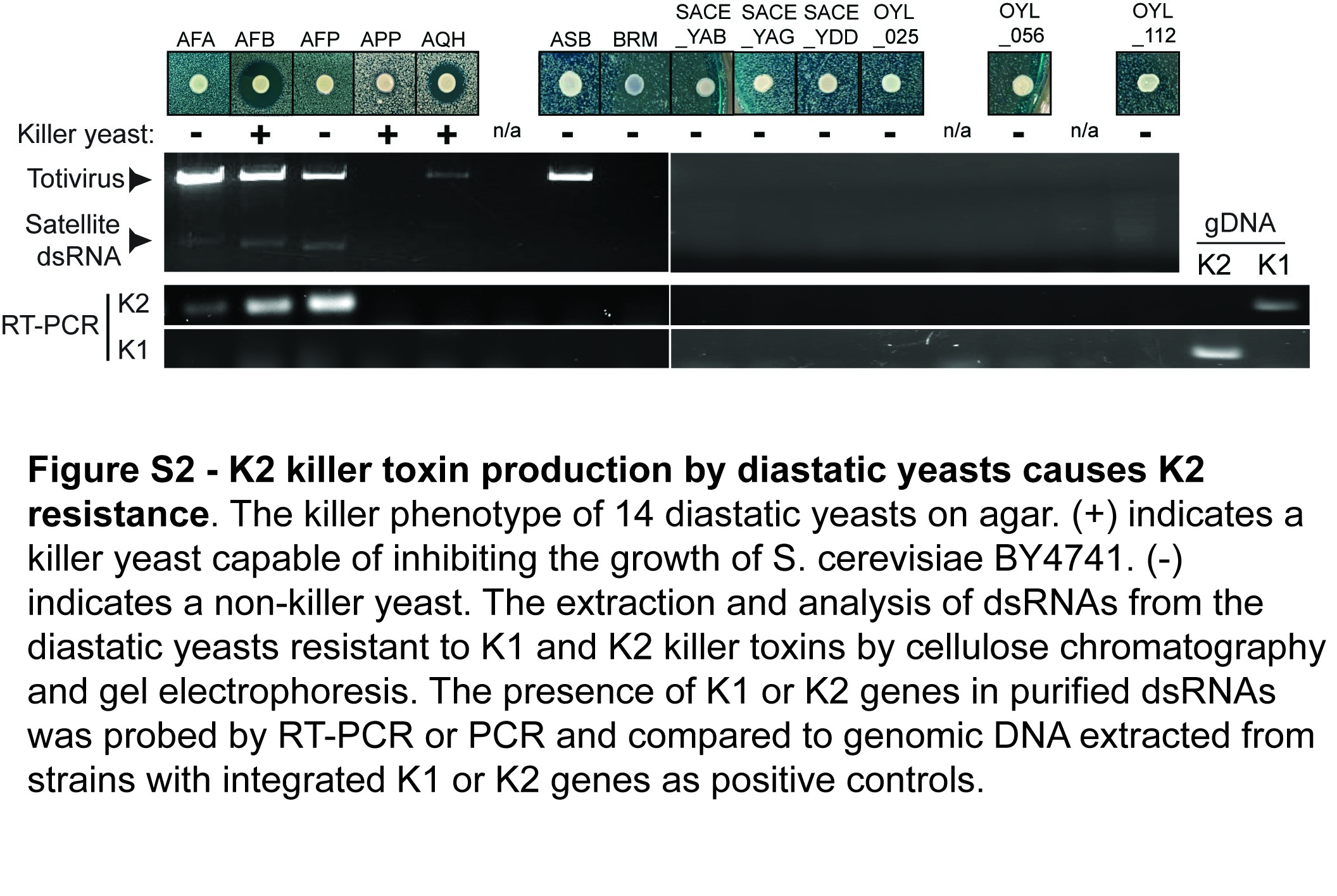

Supplement: Figure S1 — K2 killer toxin production by diastatic yeasts causes K2 resistance. [file aem.01072-24-s0002.tif]
